# Evaluation of Physics-Based Pose Prediction and the Role of Receptor Flexibility in a Community Antiviral Blind Challenge

**DOI:** 10.1021/acs.jcim.6c02582

**Published:** 2026-10-14

**Authors:** Asma Feriel Khoualdi, Finlay Clark, Joshua T. Horton, Daniel J. Cole

**Affiliations:** † School of Natural and Environmental Sciences, 5994 Newcastle University, Newcastle upon Tyne NE1 7RU, U.K.; ‡ Open Free Energy, Open Molecular Software Foundation, Davis, California 95616, United States

## Abstract

Prediction of protein–ligand binding modes is a key step in computer-aided drug design, since incorrect pose prediction inevitably limits the achievable accuracy of downstream physics-based affinity predictions. The ASAP-Polaris-OpenADMET antiviral competition was launched in 2025 as a means to prospectively test computational methods across a range of measurements that guide early prioritization during a drug discovery campaign including pose prediction. Here, we describe our two open-source, physics-based entries to the pose prediction challenge, which are based on templated molecular growth with hybrid machine learning/molecular mechanics interatomic potentials. We show that our approach was among the top-performing physics-based methods, and with a retrospective change to the workflow to make greater use of the training data as templates, we obtain a success rate of 74% across SARS-CoV-2 and MERS-CoV main protease target protein structures. Finally, we investigate the importance of receptor side-chain flexibility on retrospective performance. We find that building poses against an ensemble of feasible side-chain conformers is useful in generating improved binding poses but that the tested scoring functions are unable to successfully pick out native structures from the generated ensemble.

## Graphical abstract

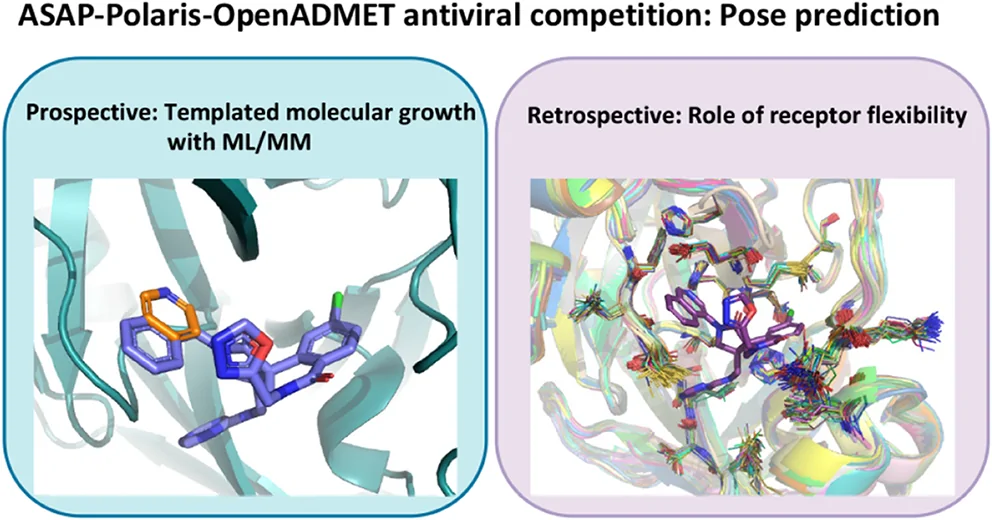

## Introduction

The drug discovery process is arduous, time-consuming, and expensive, often requiring over a decade of research and billions in investment to bring a single therapeutic to market.^1^ Traditional experimental screening approaches are labor-intensive and resource-intensive, which limits their utility in rapidly responding to emerging health crises. Computational methods offer a supplementary approach by enabling the rapid exploration of chemical space, prioritization of promising candidates, and a reduction of experimental burden. Pose prediction is a central component of many physics-based workflows, as the accuracy of predicted ligand binding conformations directly impacts downstream analyses such as binding affinity estimation.^2,3^ Reliable poses ensure that key intermolecular interactions, such as hydrogen bonds, hydrophobic contacts, and electrostatic complementarities, are correctly captured, which is essential for meaningful scoring.^4^ Moreover, high-quality pose prediction is crucial for rigorous methods such as alchemical free energy calculations, where even small structural inaccuracies can lead to significant errors in predicted thermodynamic quantities.^5–7^ Therefore, improving pose prediction accuracy not only enhances the reliability of docking-based screening but also strengthens the predictive power of more computationally intensive approaches used in hit-to-lead optimization.

FEgrow^8^ was designed as an open-source solution to the problem of pose prediction. FEgrow builds a set of congeneric molecules into the receptor-binding pocket from a defined, rigid ligand core, thereby aiming to maximize the use of crystallographic information. The total energy of the system is calculated using hybrid machine learning/molecular mechanics (ML/MM),^9^ in which the internal energy of the ligand is calculated using a machine learning potential (in this case ANI-2x^10,11^ ) and intermolecular interactions with the protein are calculated using a standard classical force field. In this way, crucial ligand conformational energetics are modeled at an accuracy approaching that of quantum mechanics at a small fraction of the computational cost. If desired, low-energy conformers can be scored using external docking scoring functions or used as input for more rigorous affinity predictions, such as free energy calculations.^8^

We have shown that FEgrow can be used to build protein–ligand complexes as input for either scoring functions or free energy calculations, with reasonable success in ranking compounds relative to experiment.^8^ The building-scoring-design cycle may be further automated using active learning,^12^ reducing reliance on human input. FEgrow also formed the basis of our submission to the CACHE Challenge 2, in which we prospectively designed a pyrazole-based hit series targeting the RNA site of the SARS-CoV-2 helicase Nsp13.^13^ We have found that community-blind challenges such as these are invaluable in providing an objective comparison between different computational methods. These challenges establish a fair benchmark, as all participants use the same data, rules, and evaluation metrics. They are important not only because they help identify the method that works best but also because they discern areas where all methods struggle and where further development effort is needed. Other prominent examples of blind challenges include the Drug Design Data Resource (D3R) challenge, which is aimed at identifying the best computer-aided design method through blinded ligand pose prediction and affinity challenges^14^ and the Critical Assessment of Structure Prediction (CASP) challenge, which is a competition to predict protein structure from its amino acid sequence.^15^

Here, we present details of our entry to the pose prediction challenge of the ASAP-Polaris-OpenADMET antiviral competition (henceforth referred to as the ASAP blind challenge).^16^ The challenge is based around data collected by the AI-driven Structure-enabled Antiviral Platform Discovery Consortium (ASAP) as part of an open-science initiative to design pan-coronavirus (SARS-CoV-2 and MERS-CoV) main protease (Mpro) inhibitors. Mpro is a cysteine protease essential for viral replication and an attractive therapeutic target.^17^ Its role in the COVID-19 pandemic attracted many drug design efforts, with some approved inhibitors, including Nirmatrelvir (Paxlovid)^18^ and Iscartrelvir.^19,20^ The ASAP initiative itself builds on the success of the COVID Moonshot consortium in demonstrating the feasibility of an open-science approach to Mpro inhibitor design during the COVID-19 pandemic.^21^ The ASAP blind challenge was launched in 2025, in collaboration with Polaris^22^ and OpenADMET,^23^ and attracted 381 challenge submissions across 66 unique participants. In contrast to many other challenges, the ASAP-Polaris-OpenADMET antiviral competition used withheld “real-world” data to challenge participants, focused on one target from one experimental source, using a training/test time split, and considered measurements that guide early prioritization during a drug discovery campaign. The challenge served as a benchmark for current methods in drug discovery, and its held-back data allowed the measurement of prospective accuracy. The challenge was performed via three competition tracks: antiviral ligand poses, potency, and Absorption, Distribution, Metabolism, Excretion, and Toxicity (ADMET). In the ligand pose prediction category that we took part in, participants were provided with 770 SARS-CoV-2 Mpro crystal structures as a training set and asked to predict the binding poses of the test set of 98 SARS-CoV-2 Mpro ligands and 97 MERS-CoV Mpro ligands. For evaluation, the number of poses with lower than 2 Å symmetry-corrected heavy-atom root-mean-square deviation (RMSD) (when measured against the blinded experimental crystal structure poses) was used as the success metric.^24^

Pose-building entries to the challenge were classified into three main categories:^16^ deep-learning docking, physics-based, and cofolding. The top-performing category was found to be the cofolding methods, in particular based on AlphaFold3 ^25^ and Boltz-1 ^26^ architectures, with several entries achieving >80% success rates.^16^ One major disadvantage of such methods is that cofolding methods can struggle to generalize beyond the training data, and it is likely that Mpro structures are well represented in the models’ training data.^27^ Indeed, a follow-up ligand-only baseline, based on constrained 3D embedding to available reference ligands, achieved a high success rate of around 70%, highlighting the good availability of closely related ligand poses.^28^ An alternative to cofolding approaches, where the receptor structure is known but data are otherwise scarce, might be deep-learning-based docking methods. Methods classified by the organizers in this category showed the worst performance out of all the methods used (0–29% success rate). However, it should be noted that at least one method classified as cofolding did use pose refinement with deep-learning-based docking, and so workflows that combine multiple categories of methods may also be of value.

In any case, even though cofolding methods performed well in this competition, it is still of considerable interest to drive physics-based solutions for new targets with low data availability. Physics-based models can even be used to expand the training data for cofolding and other models.^29^ There were 13 entries classified by the organizers as physics-based methods, with success rates ranging from 16–67%. The top-scoring method was the baseline method used by the organizers, which cross-docked each input molecule to the five closest available structures in the training set using OpenEye POSIT,^30^ and the highest-scoring pose was selected based on the ChemGauss4 score.^31^ The next-best physics-based entry used FlexX^32^ for template docking into the complex with the highest maximum common substructure (MCS) similarity to the test ligand, achieving a score of 62%. Other traditional docking approaches that did not perform as well include Glide^33^ and an EasyDock/AutoDock pipeline.^34,35^ However, one protocol that did achieve >50% success rate combined Vina with fragment-constrained pose generation (that is, similar to the default FEgrow workflow, identified fragments are restrained in their crystallographic poses).^36^ An ablation study by the same authors identified the importance of a custom fragment overlap scoring function and of building poses against an ensemble of receptor conformations drawn from crystallographic structures and a Monte Carlo side-chain ensemble computational approach.^37^

As we report here, our fragment-constrained approach with FEgrow was competitive with those of many of these physics-based methods. However, on analysis of our competition entry, it became clear that not considering side-chain flexibility was a limiting factor in the performance of physics-based approaches with rigid receptors (see Results section), despite the previously reported rigidity of the SARS-CoV-2 Mpro binding pocket. Here, we therefore *retrospectively* investigate how we might combine FEgrow with a method for sampling the conformational space of flexible side chains and subsequently score the resulting complexes to recover the native pose.

There are a number of computational methods available for generating ensembles of feasible protein side-chain conformations. The first class, as already mentioned, is the cofolding approaches, such as AlphaFold,^25^ Boltz,^38^ Chai,^39^ and OpenFold.^40^ Methods such as these, in the best cases, are capable of predicting ligand, backbone, and side-chain conformers to high accuracy, but as discussed generally rely on the availability of close homologues in the training data.^41^ To constrain the conformational search space, we focus here instead on methods to generate side-chain ensembles given a known backbone structure. DLPacker^42^ uses deep neural networks to predict side-chain geometry, with improved accuracy compared to physics-based methods. AttnPacker^43^ utilizes an SE(3)-equivariant neural network architecture to simultaneously predict all side chains and bypasses the need for a discrete rotamer library, which omits expensive conformational search and sampling steps. While these methods provide a solution to side-chain packing, their predictions are ligand-agnostic, which may hinder the generation of suitable holo-binding pockets for ligand posing. Instead, we have utilized here Apodock,^44^ an open-source software that leverages both 2D ligand information as well as the 3D backbone structure to generate ensembles of plausible side-chain structures.

In this work, we analyze our original ASAP pose prediction submission and attempt to retrospectively improve performance by sampling varied pocket conformations using Apodock. Pose prediction using receptor ensembles requires consideration of two factors—generation of high-quality poses and prediction of the native crystallographic pose from the generated ensemble. To address the latter issue, we evaluate the ability of deep learning-based scoring functions (ApoScore^44^ and gnina^45^), as well as more computationally costly absolute binding free energy (ABFE) calculations,^46^ to select high-quality poses from a pool of structures. We show that it is indeed feasible to obtain a good success rate by building ligand poses against a single receptor structure, but further work is required to improve the scoring functions so that the correct pose is identified.

## Methods

Pose predictions for both our prospective submissions to the ASAP challenge were determined using the FEgrow software, with standard settings.^8^ Namely, for each ligand, an ensemble of 500 conformations was generated using the ETKDG algorithm^47^ in RDKit and preoptimized with the Universal Force Field^48^ while applying a tight harmonic restraint (10^4^ kcal mol^–1^ Å^–2^) to the user-defined core. Conformers within 0.5 Å RMSD of an existing conformer or with close clashes (any atom–atom distance less than 1 Å) with the protein were discarded. The remaining ensemble (typically <100 conformers per ligand) was optimized using OpenMM,^49^ holding the protein positions fixed. AMBER FF14SB^50^ was used to parametrize the protein, and OpenFF-Sage^51^ with AM1-BCC charges^52^ from AmberTools was used to represent the nonbonded terms of the target ligand. Following our default protocol, Lennard-Jones radii and atomic charges were scaled down during optimization to mitigate to some extent the rigid-protein approximation.^8^ Ligand intramolecular energetics were described using the ANI-2x machine learning potential.^11^ After energy-minimizing each of the conformers with the hybrid ML/MM method, the lowest-energy conformation was extracted as the best pose.

### Prospective Submission: Manual Core Selection

The ASAP pose prediction challenge organizers provided 770 protein–ligand complexes of SARS-CoV-2 Mpro as training data and with a time limit of 8 weeks asked us to predict the poses of 98 ligands (the “test set”), aligned to a provided reference structure. In this entry, we only used the training data to define the fixed, common core structure from which to grow the remaining 98 structures. The core was selected as a pyridine moiety, which is conserved across 96 of the 98 SARS-CoV-2 Mpro test set molecules. With hindsight, we could have alternatively chosen an isoquinoline core to break the core symmetry and better define the exit vector, but in practice we observed that all compounds were grown into the expected binding pocket. The core was taken from complex-79 from the training set after alignment to the provided reference structure, as, by inspection, it provided a well-conserved vector into the binding pocket (Figure 1a). The protein structure used in FEgrow was the reference structure provided by the organizers, and all protonation states were retained from those provided. Since eight structures could not be built into the receptor, a different receptor (from complex-48) was selected to grow these molecules, as it had some movement in key residues, such as Q189 and M49, in the binding site. The remaining four SARS-CoV-2 Mpro test set structures were finally docked against the reference receptor structure using the gnina docking software,^45^ with the provided ligand reference pose to define the center of the box, which was of the default size.

**Figure 1:**
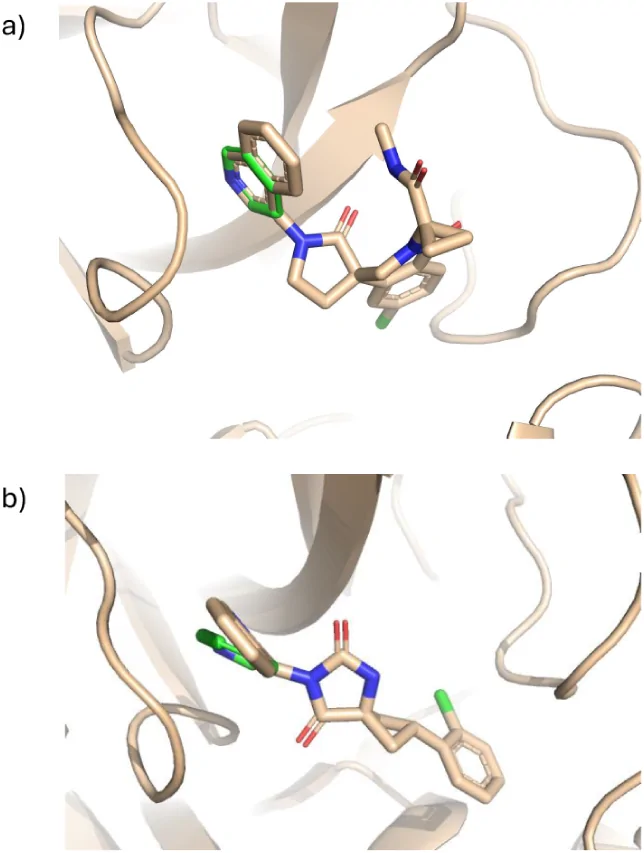
(a) The pyridine core (green) overlaid on complex-79 from the SARS-CoV-2 Mpro training data (gold). (b) The pyridine core (green) overlaid on the provided MERS-CoV Mpro reference structure (gold).

For the MERS-CoV Mpro set, the organizers only provided a single reference complex structure and challenged us to build a provided test set of 97 ligand structures into the binding pocket. Upon inspection of the reference structure, the isoquinoline moiety of the provided ligand structure appeared to be distorted (Figure 1b). To confirm this, we removed the isoquinoline substructure of the ligand and rebuilt it using FEgrow. The resulting pyridine core was used as the common scaffold for growing the remaining ligands (Figure 1b). With this approach, seven molecules could not be built. As an alternative strategy, we aligned complex-79 from the SARS-CoV-2 training set with the MERS-CoV reference structure and extracted the pyridine core for use as a scaffold to build these molecules. Finally, the remaining four molecules that could not be built with FEgrow were docked using gnina, as above.

### Prospective Submission: Maximum Common Substructure Core Selection

In a second, separate submission, we aimed to make the choice of core less arbitrary and to instead select it from the training data using a maximum common substructure (MCS) search. In this way, we increased the amount of information extracted from the crystal structure data provided. Since many reference receptor structures were available for SARS-CoV-2 Mpro, we extracted the ten cores from the training set with the highest MCS overlap, measured using RDKit,^53^ for each test ligand. We then attempted to build the test set ligand using each core in turn (in order of decreasing MCS overlap) into the provided reference protein structure, terminating the procedure once a structure was successfully built. From this automated procedure, 14 molecules failed to be built successfully. For these molecules, a second run was attempted using OpenFF-Sage^51^ for ligand intramolecular energetics, replacing the ANI-2x machine-learning potential. Finally, for molecules that still could not be built, they were replaced with their counterparts from our first prospective submission. For MERS-CoV, only a single training-set structure was provided (the reference structure), so the core was extracted from this structure for all test molecules (although MCS was still used to determine the size of the core for each test ligand). Note that we first used FEgrow to rebuild the pyridine group, as in Figure 1b.

Upon the release of the results from the prospective challenge, it was clear that we had incorrectly aligned 11 of the structures submitted using the MCS alignment method to the wrong chain of the receptor. This was symptomized by a very high RMSD relative to the native pose. In the data presented here, we have corrected the ligand alignments for these compounds and recalculated our competition metrics.

### Retrospective Analysis: Combining FEgrow and ApoDock

In order to investigate the effect of side-chain mobility on the accuracy of pose prediction, we interfaced FEgrow with the output of the ligand-conditioned side-chain packing software, ApoDock.^44^ ApoDock takes a protein backbone structure and ligand SMILES string as input, and predicts an ensemble of protein–ligand complex structures (with a rigid backbone), in a manner analogous to induced fit. In particular, the ApoPack module of ApoDock embeds ligand features as atomic properties, and the residue features are the protein sequence and 3D atom–atom distances. The features are updated through a message-passing neural network layer and used to predict side-chain torsion angles. We used the reference protein structures and ligands provided by the organizers to produce an ensemble of 20 receptor structures for each of SARS-CoV-2 and MERS-CoV Mpro, with a variety of possible flexible side-chain conformations. It has been shown that ApoPack achieves an accuracy of around 60% (RMSD < 1 Å) for side-chain packing prediction on the PDBBind data set,^54^ with this accuracy starting to saturate after around 20 generated samples.^44^ Note that for simplicity, we did not repeat the full ApoDock workflow for each provided test ligand, but it is possible that further accuracy gains are achievable by conditioning receptor side chains on a ligand-by-ligand basis. Following the generation of the 20 candidate receptor structures, we used the same methods as discussed above for the prospective submission to grow the test ligands from a common core using FEgrow. The cores used for SARS-CoV-2 Mpro and MERS-CoV Mpro are the same as those used in Figure 1. ApoScore,^44^ a mixture density network (MDN)-based scoring function, which uses ligand information, pose coordinates, and 3D pocket information, was used to rank the generated complexes. For comparison, we also evaluated the gnina convolutional neural network scoring function^45^ and fast absolute binding free energy calculations^46^ (Section S1) for pose ranking. We note that apart from the use of hindsight, we did not use any information here that was unavailable at the start of the blind competition.

## Results

### FEgrow Is among the Top-Scoring Physics-Based Methods in the Prospective Pose Challenge

The ASAP blind challenge began in January 2025 and ran for 8 weeks.^16^ At the close of the competition, the organizers released the X-ray crystal structures of all of the test-set protein–ligand complexes, as well as the competition leaderboard with entries ranked by the percentage of structures with a symmetry-corrected RMSD to the crystal structure <2 Å. Initially, our standard FEgrow submission, growing from the manually selected pyridine cores, found 47% of structures with an RMSD < 2 Å. Using MCS to automate the core search at first sight did not improve the results substantially (48% of structures with an RMSD < 2 Å), but follow-up analysis revealed that a number of entries for SARS-CoV-2 had not been aligned correctly with the reference structure, which led to very high anomalous RMSD. In the analysis below, we correct this alignment issue.

Figure 2 shows the distribution of the RMSDs of each of our submissions, now separated into SARS-CoV-2 and MERS-CoV test sets. Our manually selected core submission performed better for SARS-CoV-2 (Figure 2a) than for MERS-CoV (Figure 2b), with around 60% and 40% of structures, respectively, with an RMSD < 2 Å. This was the case for nearly all of the competition submissions and likely reflects the greater information available in the training set for SARS-CoV-2. Although we did not explicitly use the training data in our first physics-based approach with the pyridine core, the provided structures were helpful in informing the choice of core and growth vector. Also of note, in Figure 2a, the MCS-based core selection performs better than the single choice of core for SARS-CoV-2, with around 70% of structures <2 Å RMSD. This reflects the wider selection of ligand cores available in the SARS-CoV-2 training set and indicates that this approach may be useful at later project stages. This is also consistent with the observation that most of the top-scoring physics-based methods employ template alignment. Overall, after correcting for user alignment error, the MCS-based approach finds 54% of structures with <2 Å RMSD. Although still lower than the POSIT and FlexX template-based docking approaches,^16^ this would have moved our FEgrow entry alongside another template-based approach as the top-ranked open-source software entries.

**Figure 2:**
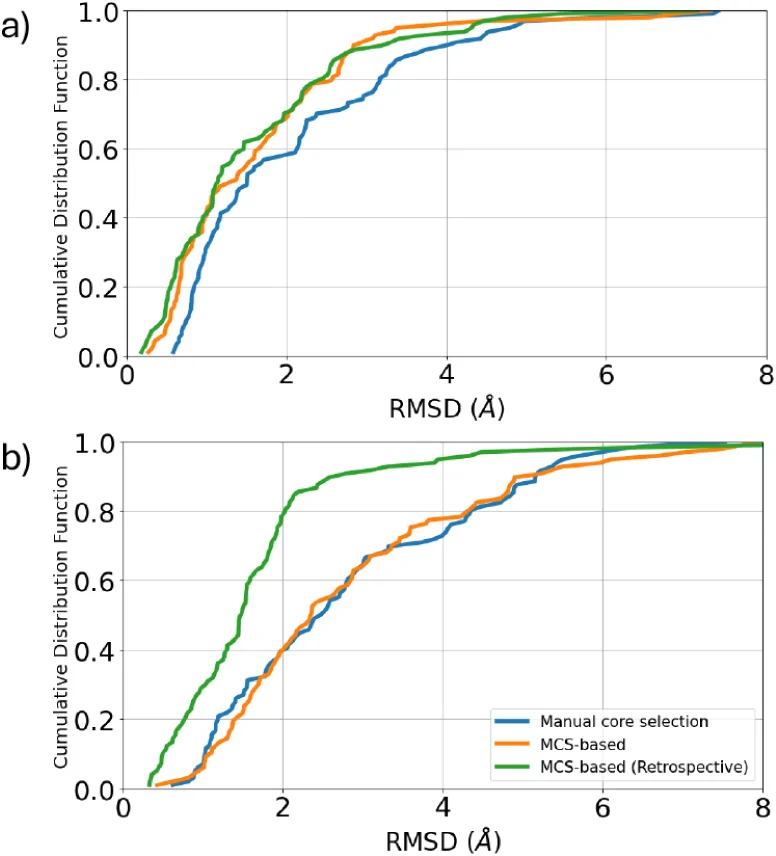
Cumulative distribution of RMSD pose errors compared to experiment of our competition entries for (a) SARS-CoV-2 and (b) MERS-CoV Mpro, for different core selection strategies.

Finally, noting the general success of template-based pose prediction and the wide use of receptor structures from the training set,^16,28^ we *retrospectively* sought to adjust our workflow to address the low success rate for MERS-CoV Mpro. In particular, we repeated the MCS-based procedure, but instead of using a single reference protein structure, we used the protein structure from the training set of the closest MCS match. That is, we hypothesize that closely related ligands will also have closely related receptor structures. Second, instead of using a single MERS-CoV reference core, we switched to using the SARS-CoV-2 training data to define both the core and protein binding pocket. Ligands were built into the SARS-CoV-2 training protein structures using FEgrow, and subsequently mapped back onto the MERS-CoV reference structure by performing a protein backbone alignment in PyMOL.^55^ This overall approach should work well if the ligand’s intrinsic low-energy conformations are more important than the structural differences between these close protein homologues. The results are shown in Figure 2 (MCS-based (retrospective)). The change in procedure does not affect accuracy for SARS-CoV-2 (around 70% of structures with <2 Å RMSD), but the results are significantly improved for MERS-CoV (around 80% of structures with <2 Å RMSD). Overall RMSD accuracy is now 74%, which would have been the competition’s top-performing physics-based method and among the best across all categories for MERS-CoV.^16^

### Rigid Receptor Approximation Limits Achievable Accuracy

Figure 3 shows some example overlays between our submitted binding poses and the released crystal structures for SARS-CoV-2 Mpro. In best-case scenarios (Figure 3a), there is an almost perfect match between prediction and experiment. Frequently, visual inspection reveals a relatively trivial torsional rotation that could be close in energy to the true structure (e.g., Figure 3b), which nevertheless causes a high measured RMSD (>2 Å). For worst-case scenarios (e.g., Figure 3c), we observe a very different predicted binding mode that would not be recoverable on the time scale of typical molecular dynamics (or free energy) simulations. Closer inspection of the released and predicted structures in this case reveals that the native binding pose would actually clash with the side chains of the reference protein structure (Q189). Thus, there is no chance that the correct binding pose would be recovered by using the provided reference structure and the rigid-receptor approximation inherent in many docking workflows.

**Figure 3:**
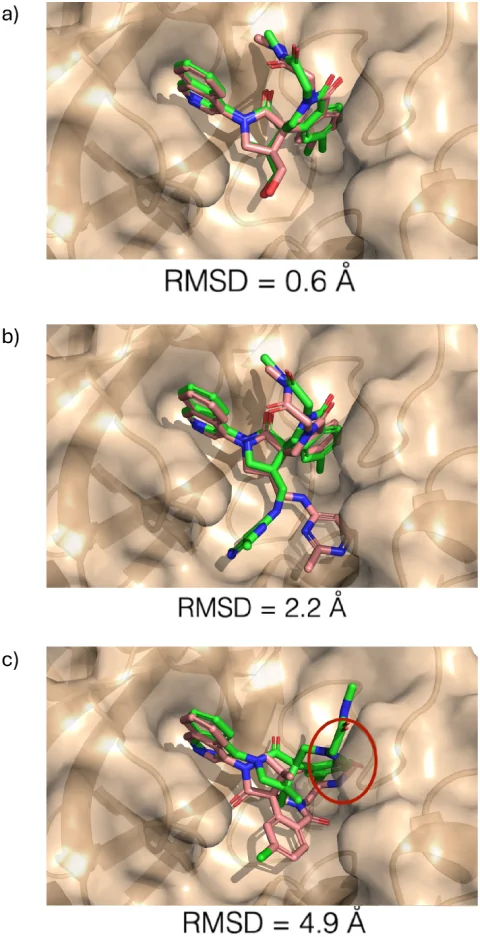
Examples where the submitted pose (colored in peach): (a) closely matches the true structure (green), (b) fits but has an incorrectly positioned moiety, and (c) has a high RMSD between the molecules due to the Q189 side chain of SARS-CoV-2 Mpro that occludes the true binding pose (circled).

Since the receptor structures for all ligands for SARS-CoV-2 and MERS-CoV have been released by the organizers, we overlaid them to scrutinize the binding pocket for any ligand-induced flexibility at this site. By inspection (Figure 4), the backbone structures are strongly conserved, but there is some flexibility in certain side chains. Namely, the M165, Q189, and M49 side chains in the SARS-CoV-2 Mpro receptor, and Q192, L49, M25, Q167, and M168 in MERS-CoV Mpro, show significant movement that could affect predicted ligand binding poses if not accounted for. This observation is further examined in Figure 5, where in addition to the RMSD of the ligand pose, we also quantify the RMSD between the side chains in the reference structure and the released crystal structures (red points). We do not observe any correlation between the ligand and pocket RMSD (that is, we cannot say that the pose prediction is more challenging when the ligand induces a large structural change in the pocket). However, the reference structure for SARS-CoV-2 is generally closer to the target structures (93% of crystal test structures have a pocket RMSD < 2 Å relative to the provided reference structure) than for MERS-CoV (32% of test structures), which may explain the overall better competition results for SARS-CoV-2.

**Figure 4:**
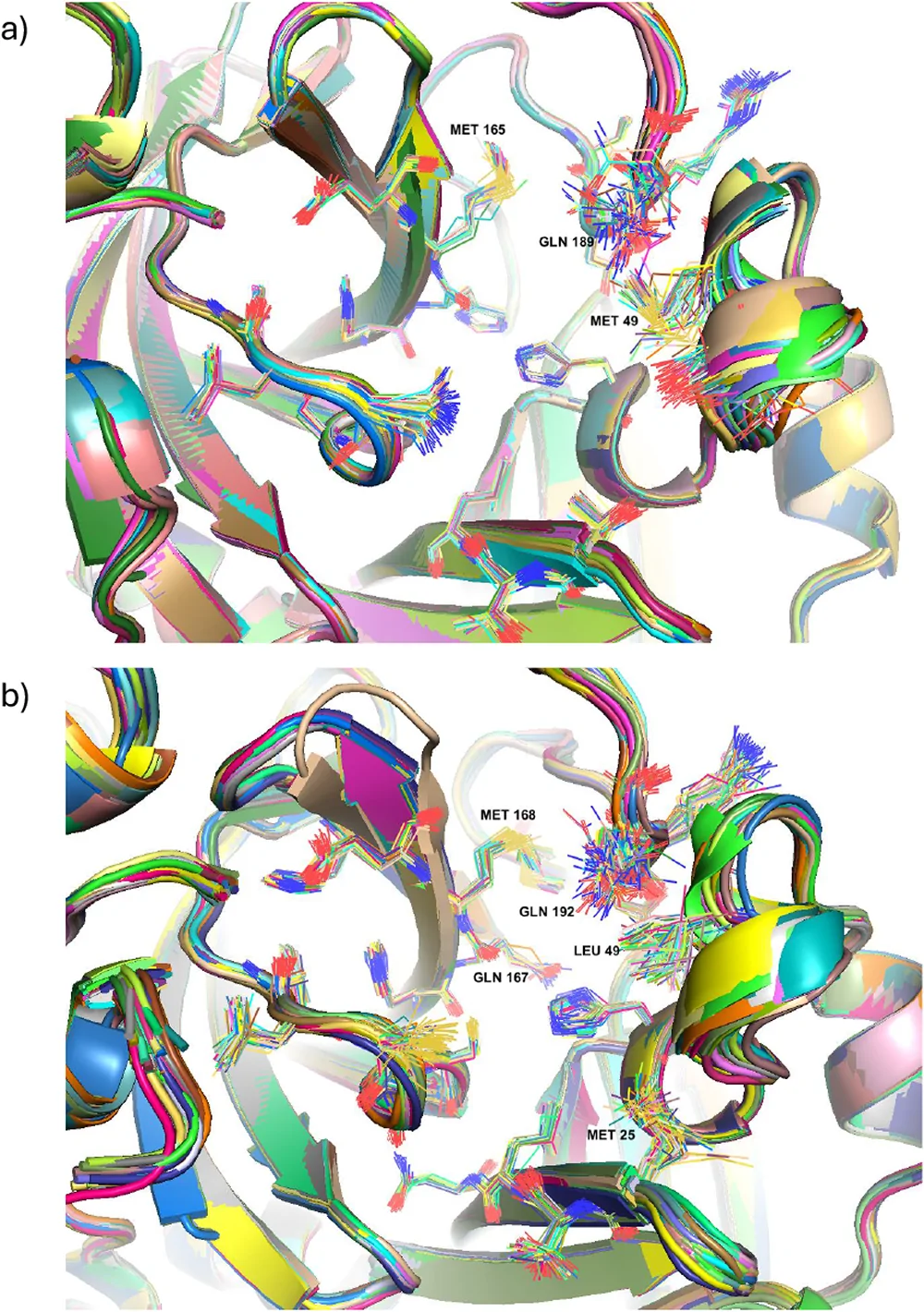
Retrospective overlay of the experimental receptor test set structures for (a) SARS-CoV-2 Mpro and (b) MERS-CoV Mpro. Significant side chain flexibility is observed for several labeled residues.

**Figure 5:**
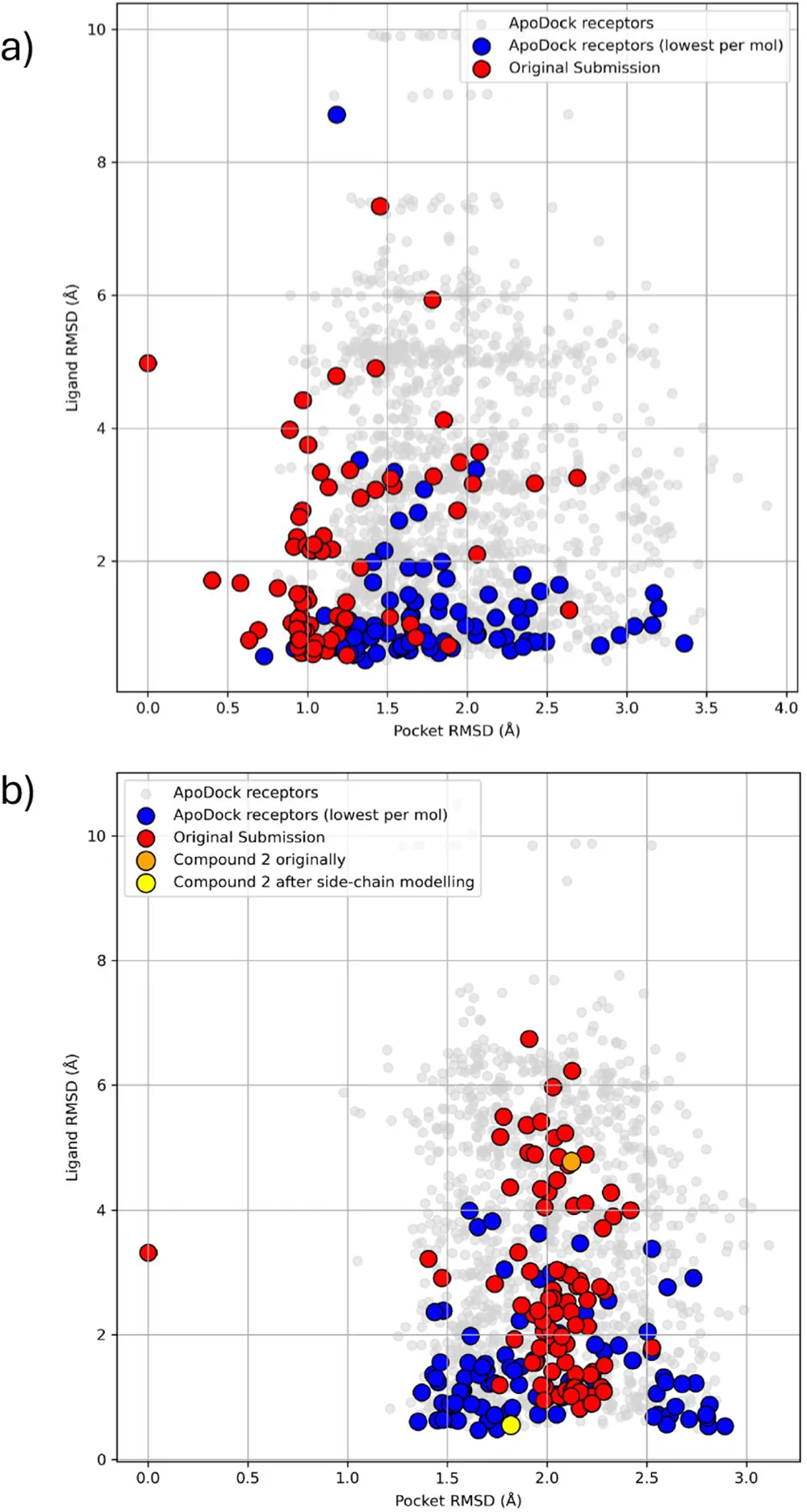
Scatter plot of ligand and pocket residue RMSDs relative to the released crystal structures for (a) SARS-CoV-2 and (b) MERS-CoV Mpro. Red circles denote data for our original competition entry. Gray circles are the ensemble of structures generated by our ApoDock/FEgrow interface. Blue circles are a subset of the gray circles, showing the lowest ligand RMSD for each test set ligand.

As discussed in the methods, we used the ligand-conditioned side-chain-packing software, ApoDock, to generate an ensemble of 20 structures with remodeled side chains for each target. We next used FEgrow to build the test sets of ligands into each of the 20 receptor structures for SARS-CoV-2 and MERS-CoV Mpro, using the core structures pictured in Figure 1. Figure 5 additionally shows all of the resulting ligand and pocket RMSDs relative to the released crystal structures (gray points), and Section S2 analyzes the corresponding protein–ligand interaction fingerprints. Again, we do not see any pattern emerging between low pocket RMSD and an accurate ligand-binding pose; however, we do observe an increase in available pocket structures with an RMSD < 2 Å relative to the released crystal structures, particularly for MERS-CoV. To quantify this effect, we narrow the selection down to the subset of structures with the lowest ligand RMSD relative to the crystal structure for each compound (shown as blue points). For SARS-CoV-2, 72% of structures in this subset have a pocket RMSD < 2 Å compared to the released experimental structures. For MERS-CoV, this fraction is 59% (increased from 32% without side-chain remodeling), which indicates that there may be some advantage to side-chain packing when dealing with a more flexible target.

Figure 6 (blue histograms) additionally shows the distribution of per-residue RMSDs of the test-set receptor crystal structures measured relative to the provided reference structures. Overlaid are the same distributions for the ApoDock-generated structures. Although not in perfect agreement, we can see that the distributions of structures produced by ApoDock are similar to those observed experimentally, at least in terms of their overall RMSD values relative to the reference structure. Residue L49 in MERS-CoV shows the greatest discrepancy between the reference and target structures experimentally, and this effect is underestimated by ApoDock, which tends to produce structures closer in RMSDs to the reference.

**Figure 6:**
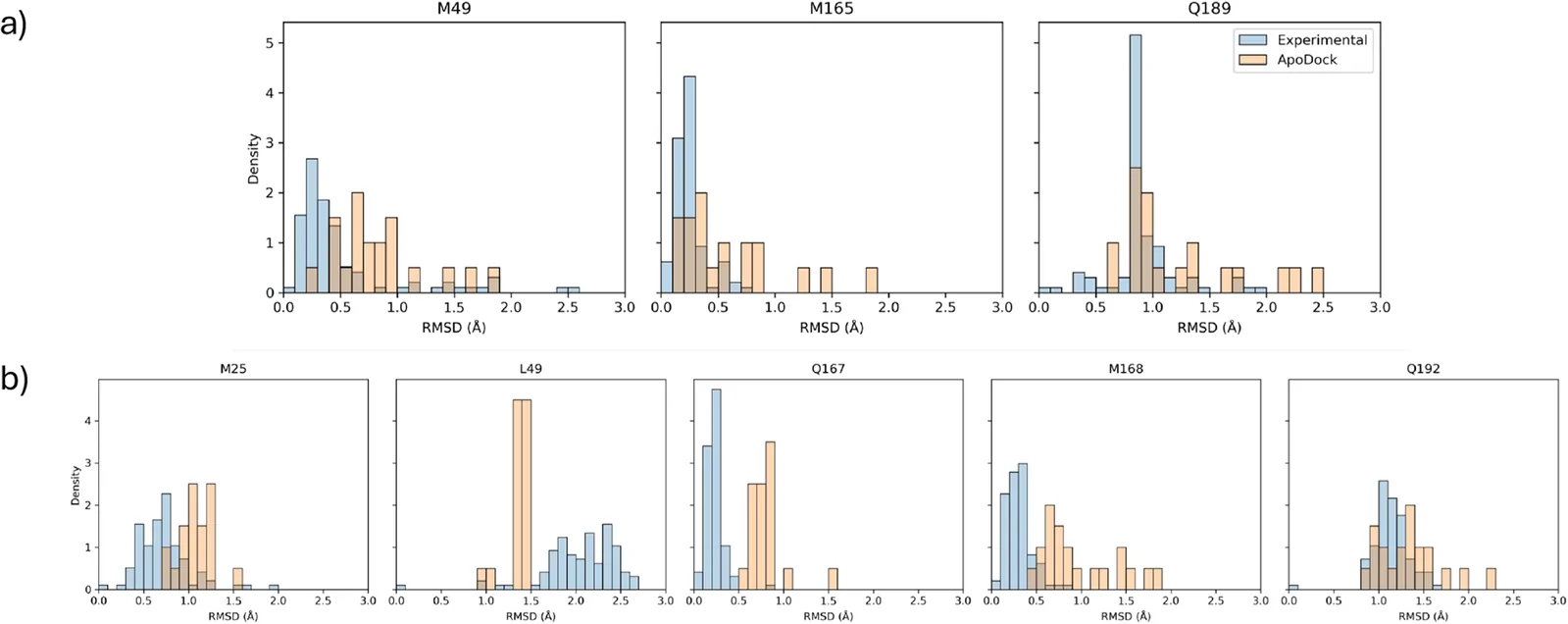
RMSD distribution (relative to the reference structures) of the modeled side chains for (a) SARS-CoV-2 Mpro and (b) MERS-CoV Mpro targets compared to the released test set crystal structures.

We illustrate here the example of compound 2 from the MERS-CoV Mpro test set, where the side chains have an essential role in guiding the ligand to the correct binding pose. Figure 7a shows an overlay of the submitted pose (peach) with the experimental crystal structure (gold). The provided reference protein structure has L49 oriented away from the binding pocket. Correspondingly, the predicted FEgrow structure twists outside the binding pocket to form close contact with L49. In contrast, the crystal structure shows an induced fit of the ligand, in which both the ligand and the side chain of L49 are buried and close-packed with each other. Figure 7b shows the same compound grown into one of the ApoDock-generated structures (pink). In this case, the side chain is optimally oriented for close packing with the ligand, and the ligand RMSD is substantially reduced to 0.5 Å, as also demonstrated by the highlighted points in Figure 5.

**Figure 7:**
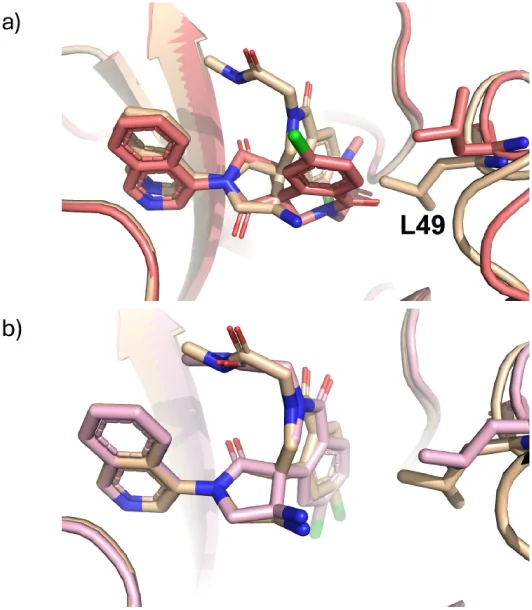
(a) Submitted pose for compound 2 of the MERS-CoV test set (peach). (b) The same ligand, but now built against a receptor structure generated by ApoDock (pink). In both cases, the crystal structure is overlaid in gold.

### Scoring Functions Cannot Reliably Sort Binding Poses from Decoys

The above analysis reveals that growing from a template structure against an ensemble of receptor structures can generate a good number of reliable binding poses with low ligand RMSD relative to the crystal structure. This is confirmed by Figure 8, where we plot (blue curve) the cumulative distribution of ligand RMSD, relative to the experimental test set structures, for the lowest-RMSD pose (top-1). Here, around 90% of SARS-CoV-2 and 80% of MERS-CoV Mpro structures are successfully recovered (ligand RMSD < 2 Å). However, this is only useful in a design setting if we have a method to *prospectively* select the top pose from the ensemble of (in this case 20) possible structures.

**Figure 8:**
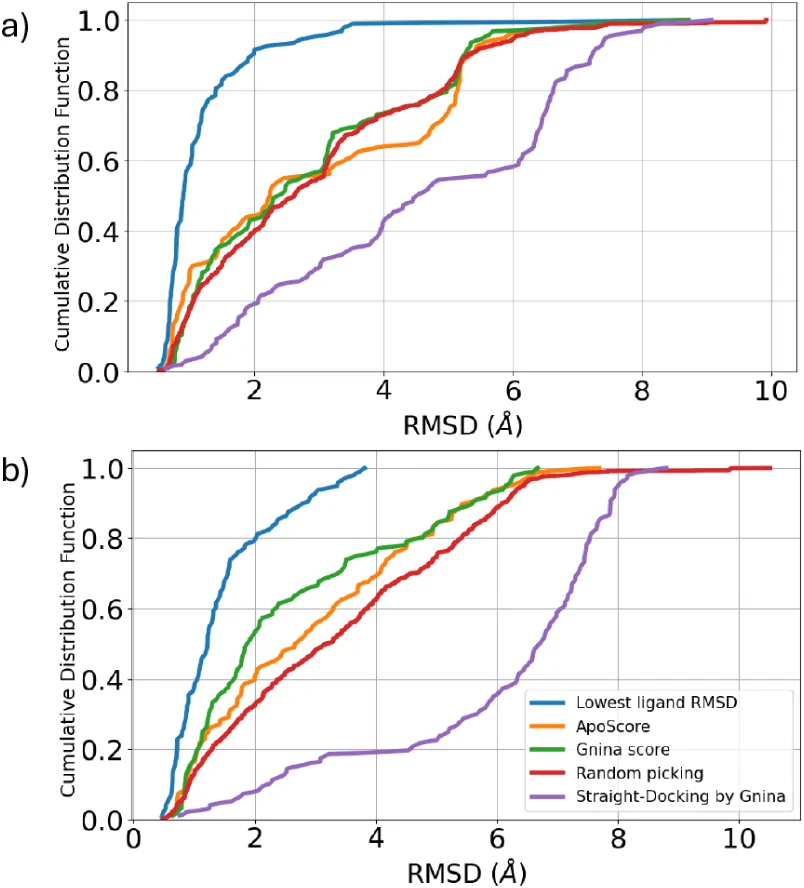
Retrospective analysis comparing different scoring functions used to pick the top-1 structure from an ensemble for (a) SARS-CoV-2 and (b) MERS-CoV Mpro data sets.

To investigate whether docking scoring functions might be used to prioritize binding poses, we postprocessed each proposed structure with the gnina and ApoScore packages, which are the default scoring functions in FEgrow and ApoDock, respectively. The resulting top-1 poses are shown in the green and orange curves in Figure 8. Also plotted for comparison is the result of picking a structure out of the ensemble at random (repeated 100 times and averaged). For SARS-CoV-2, neither scoring function is significantly better than random, with all three methods picking around 40% of structures with a ligand RMSD < 2 Å. Notably, this workflow also performed worse than our original submissions (around 60–70% of structures with a ligand RMSD < 2 Å), indicating that when we have good confidence in the structure, it is beneficial to simply build molecules into the holo crystal structure.

The message is somewhat different for the MERS-CoV Mpro structure. In this case, ensemble growing, paired with postprocessing by gnina, appears to give some benefit (around 60% of structures with a ligand RMSD < 2 Å, Figure 8b). This is now higher than our original submission, and also both ApoScore and random picking, which all identify around 40% of structures with a ligand RMSD <2 Å.

To summarize, although physics-based template growing of molecules onto an ensemble of receptor structures does seem to generate reliable binding poses, it is challenging to recover the optimal poses from the ensemble. Neither of the trialed scoring functions does much better than random picking for this task. However, we do note that this template-based approach does significantly better than an alternative baseline method, which is unbiased docking to the binding pocket. In agreement with a submission by the competition organizers, we find that docking with gnina only recovers 20% or fewer ligand poses with an RMSD < 2 Å (Figure 8, purple lines). Overall, the ensemble method slightly improves the results for MERS-CoV but worsens for SARS-CoV-2, compared to our original submission. This highlights the need to find a better way of picking out the optimal complexes from expanded pools or a better way of generating smaller pools of physically reasonable structures. With this in mind, Figure 9 shows the cumulative distribution of binding pose errors, selecting from a pool of the *N* highest-scored poses for each ligand. Again, neither scoring function is better than random picking, but encouragingly even selecting just five poses for each ligand results in at least one ligand, with an RMSD < 2 Å in around 70% of cases.

**Figure 9:**
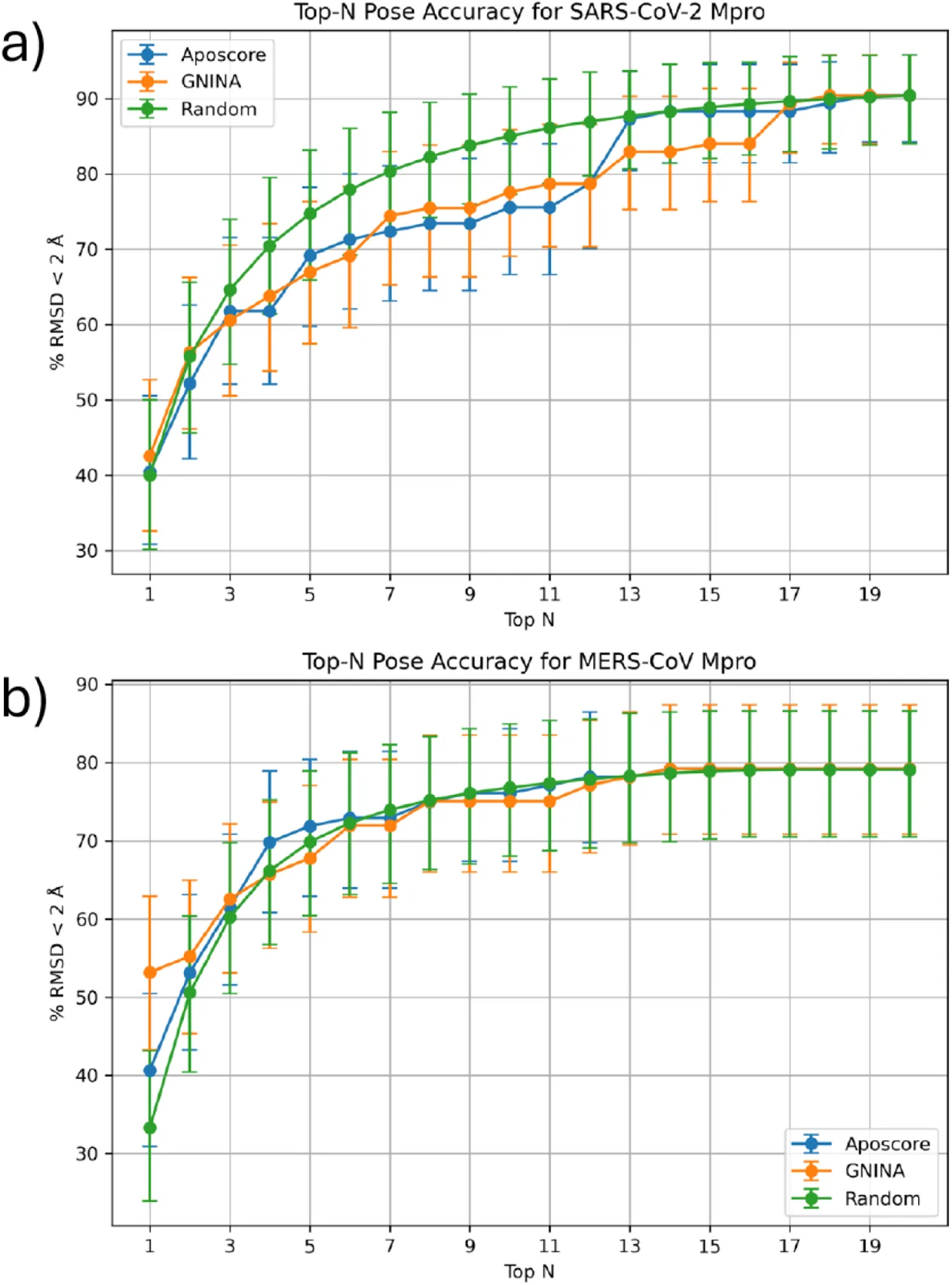
Percentage of ligands for which a pose with RMSD < 2 Å is present in a pool of the top-N scored poses across different scoring methods for (a) SARS-CoV-2 and (b) MERS-CoV Mpro data sets. Error bars represent 95% bootstrap confidence intervals.

Given that we can generate relatively small pools of receptor–ligand complexes, containing poses close to the experimental structure, we sought to understand whether a more computationally costly scoring method could discriminate between them. In particular, we assessed the ability of short (0.1 ns/window) absolute binding free energy (ABFE) calculations to recover the native poses (see Section S1 for detailed methods). Short binding free energy calculations have been shown to retain useful performance while substantially reducing computational cost,^56–58^ though it is challenging to predict how sampling time will affect performance for a given system. While ABFE calculations are most often used to predict the affinities of structurally dissimilar ligands, they can also be used to predict the preferred binding poses of individual ligands.^59,60^ We ran five independently equilibrated ABFE calculations starting from each complex for a subset of 27 neutral MERS-CoV Mpro ligands. Rigorously, the sampled configurations for each calculation should be restricted to those corresponding to the intended pose; otherwise, the resulting free energy has no clear meaning. We do not enforce this but rather rely on the short simulation time to keep the ligands kinetically trapped in a region of low RMSD compared to the initial structures. In practice, we note that there are sometimes substantial shifts, although repeating the analysis with the equilibrated pose produced equivalent results.

The mean intercomplex spread in predicted free energies per ligand (14.7 kcal mol^–1^) was substantially greater than the mean inter-replicate standard error for a single complex (1.5 kcal mol^–1^), meaning that predictions were not completely dominated by inter-replicate noise. However, the short ABFE calculations were unable to recover the lowest-RMSD conformers better than random picking; using a paired bootstrap procedure with resampling over ligands, there was no evidence for a significant improvement with the ABFE scores compared to random picking at the 2 Å threshold (Figure 10). We also assessed the ability to recover low-RMSD poses using Bayly’s fraction of best ligand (FBL) metric. Specifically, we calculated the fraction of lowest-RMSD poses present in the same fraction of lowest predicted binding free energy poses, averaged from one pose up to half of the poses for each ligand.^61^ Again, there was no evidence (from a paired bootstrapping procedure with resampling over ligands) for an improved FBL with ABFE scores compared to random picking. The failure of ABFE to enrich low ligand RMSD samples could be due to a number of reasons. There may simply be too much error from insufficient sampling to yield useful free-energy predictions. For example, ligand and side-chain strain may not have been accounted for in the calculations as a result of slow relaxation of relevant torsional degrees of freedom.

**Figure 10:**
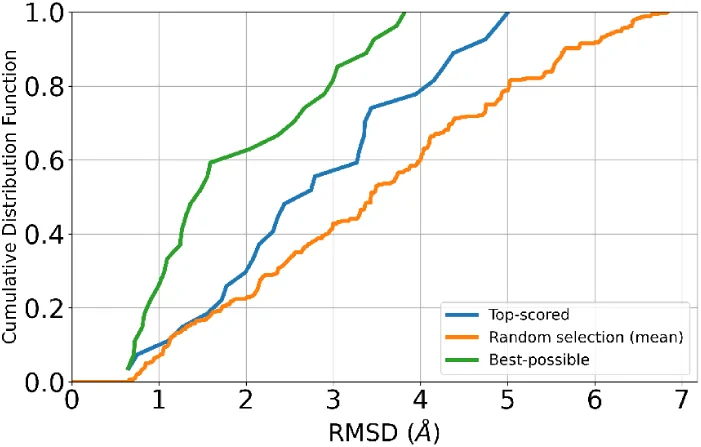
Cumulative distribution plot of ligand RMSDs for a subset of the MERS-CoV test set, showing randomly picked ligands (averaged over random picking, “Random selection (mean)”), the ligands with the top ABFE scores (lowest predicted affinities, “Top-scored”), and the best possible ligands (by ligand RMSD, “Best possible”). Using a paired bootstrap procedure with resampling over ligands, there was no evidence for a significant improvement using the ABFE scores compared to random picking at the 2 Å threshold.

## Discussion and Conclusions

We have presented here analyses of our prospective and retrospective attempts at the pose-prediction challenge of the ASAP-Polaris-OpenADMET antiviral competition. Using our open-source FEgrow software,^8^ which is based on the templated growth of molecules constrained to identified crystallographic cores using hybrid ML/MM potential energy functions, our prospective entry was ranked among the top physics-based submissions. Using an MCS-based approach to automatically identify the constrained core from the provided structural training data yielded a higher success rate (54%) than our manual core selection (47%), but it relies on sufficient training data, which may not be available early in many drug-discovery campaigns. Retrospectively, taking this approach a step further and additionally including the receptor structures from the training set and SARS-CoV-2 training data when building MERS-CoV structures yielded substantial further improvement in the success rate (74%). Together, these results indicate that the wide availability of training data benefits not only cofolding models but also physics-based models when relying on templated docking or molecular growth.

We also noted cases where it was impossible to obtain the native binding pose using a single rigid receptor, as is commonly attempted in many computational workflows. As described above, building the molecule against the receptor structure of the closest MCS match to the ligand of interest yields substantial benefits, but such data are not always available. As such, we attempted to ascertain whether it is possible to instead obtain similar results by building from a single receptor structure but using the open-source ApoDock software^44^ to generate an ensemble of plausible side-chain conformations. We showed (Figure 5) that the number of low-RMSD binding poses is enriched when building against an ensemble of receptor structures, especially for MERS-CoV, but we now faced the additional challenge of how to select the top pose from the generated ensemble (a problem also faced by cofolding methods that generate an ensemble of plausible structures). Unfortunately, we found that none of the tested scoring functions were significantly better than random picking for this task. As well as the well-publicized challenges of scoring ligand poses, an additional issue may be that scoring functions neglect the relative protein energetics. In theory, ABFE calculations provide the most physically rigorous method for scoring the thermodynamically favored complex conformation, as they make no assumptions beyond the accuracy of the force field and completeness of sampling.^62^ However, the fast ABFE calculations (necessitated here by available computational time) did not provide a useful ranking, and further work is required to score the generated poses. We hope that the open-source tools used and lessons learned here are useful to the community for further prospective drug-design tasks.

## Data and Software Availability

Our prospective challenge submissions can be found at https://github.com/cole-group/polaris-fegrow and https://github.com/jthorton/polaris_fegrow_mcs, and our retrospective data at https://github.com/AsmaFeriel000/ASAP-polaris-data. FEgrow is freely available at https://github.com/cole-group/FEgrow. The original ApoDock code may be obtained from https://github.com/ld139/ApoDock_public. The version used here (containing minor changes to facilitate installation) may be found at https://github.com/AsmaFeriel000/ApoDock_public. Challenge data released by organizers of the ASAP-Polaris-OpenADMET antiviral competition may be found at https://zenodo.org/records/15582067.

## Supplementary Material



## Notes

Methods for fast ABFE calculations and protein–ligand interaction fingerprint analysis (PDF)
