# Supplementary material for: Evaluation of Physics-Based Pose Prediction and the Role of Receptor Flexibility in a Community Antiviral Blind Challenge
Source: J Chem Inf Model. 2026 Oct 14;66(20):13597–608. doi: 10.1021/acs.jcim.6c02582 (PMC13715513; doi:10.1021/acs.jcim.6c02582)
Supplement: Supplementary file 1 [file ci6c02582_si_001.pdf]

Supplementary Information: Evaluation of Physics-Based Pose Prediction and the Role of Receptor Flexibility in a Community Antiviral Blind Challenge

Asma Feriel Khoualdi,† Finlay Clark,† Joshua T. Horton,‡ and Daniel J. Cole*,†

†*School of Natural and Environmental Sciences, Newcastle University, Newcastle upon Tyne NE1 7RU, United Kingdom*

‡*Open Free Energy, Open Molecular Software Foundation, Davis, California 95616, United States*

E-mail: daniel.cole@ncl.ac.uk

S1 ABFE Calculation Methods

Short ABFE calculations (0.1 ns /window) were run with A3FE 0.4.0 for all ApoDock-generated receptors for 27 MERS-CoV Mpro ligands.[1–6] These were the first 27 neutral ligands in the set for which all calculations completed without failures due to HPC issues. A total of 401 ABFE calculations were performed.

To prevent simulation instabilities, side chain coordinates were only taken from ApoDock structures for "binding site residues" (defined here as MET25 A, THR26 A, LEU27 A, HIS41 A, LEU49 A, PHE143 A, LEU144 A, CYS145 A, GLY146 A, SER147 A, CYS148 A, HIS166 A, GLN167 A, MET168 A, GLU169 A, LEU170 A, ALA171 A, HIS175 A, MET184 A, MET189 A, ASP190 A, LYS191 A, GLN192 A, VAL193 A, SER1 B), otherwise the side-chain coordinates from the provided structure were used.

All calculation details were the same as for the non-adaptive protocol in Clark et al. unless otherwise specified.[1] Protein and waters were parameterised with the AMBER ff14SB and TIP3P force fields,[7–9] while ligands were parameterized with OpenFF Sage 2.2.1 and AM1-BCC partial charges assigned using antechamber from `ambertools` 24.8.[10–12] Compared to the non-adaptive protocol of Clark et al., the maximum number of minimization steps was increased to 90,000 to allow any side-chain clashes from the the ApoDock structures to relax. The restrained and unrestrained NPT equilibration stages were shortened to 50 ps each, and the "ensemble equilibration" time to 100 ps. The default λ schedule in A3FE 0.4.0 was used (4 and 20 windows for the free discharge and vanish stages, respectively, and 2, 5, and 20 windows for the bound restrain, discharge, and vanish stages, respectively), which was optimized for the MIF/MIF180 system from Clark et al. with the automated window spacing algorithm described there, with a thermodynamic speed of 1 kcal mol^{-1}.[1] Concretely, windows at $\lambda = 0.0$ and 0.1 were used for the bound restraining stage; at 0.0, 0.291, 0.54, 0.776, and 1.0 for the bound discharge stage; at 0.0, 0.026, 0.054, 0.083, 0.111, 0.14, 0.173, 0.208, 0.247, 0.286, 0.329, 0.373, 0.417, 0.467, 0.514, 0.564, 0.623, 0.696, 0.833, and 1.0 for the bound vanish stage; at 0.0, 0.222, 0.447, 0.713, and 1.0 for the free discharge stage; and at 0.0, 0.026, 0.055, 0.09, 0.126, 0.164, 0.202, 0.239, 0.276, 0.314, 0.354, 0.396, 0.437, 0.478, 0.518, 0.559, 0.606, 0.668, 0.762, and 1.0, for the free vanish stage. Window spacing optimization was not repeated for each ligand in order to minimize simulation cost. Each window was simulated for 0.1 ns and the first 0.02 ns was discarded prior to analysis with PyMBAR 3.1.1.[13] 5 repeat calculations were run for each ligand: receptor pair and errors are reported as the standard error of the mean. The complete set of ABFE calculations ran in ≈ 1200 GPU-hours on Nvidia L40S GPUs (≈ 3 hours per calculation including 5 replicates). The RMSD associated with each ABFE calculation was calculated using the input ligand pose from FEGrow,[14] and it was assumed that no substantial rearrangement of the ligand occurred during the simulations so that each ABFE could be roughly assigned to each input "pose". However, rigorously, configuration space should be partitioned into volumes corresponding to each pose and each calculation should be confined to a given volume (for example, by means of restraints).

S2 Protein–Ligand Interaction Fingerprints

For each of the released experimental X-ray structures, the ProLIF software[15] was used to record the identities of all hydrogen bond donor/acceptor (using the implicit hydrogen bond method), π-stacking and anionic/cationic protein–ligand interactions present. An example is shown in Figure S1. For each ligand, the same interactions were computed for each of the 20 ApoDock-based protein-ligand complexes, and the fraction of native X-ray contacts was computed. Figure S2 plots this quantity for each of the closest matches to the X-ray crystal structure. The first point to note is that there are few of these polar contacts present for this set of ligands (maximum four), which is consistent with the hydrophobic nature of the target. Secondly, the majority of contacts is present in at least one of the computationally generated protein–ligand complexes, consistent with the generation of low-RMSD pocket conformations shown in Figure 5.

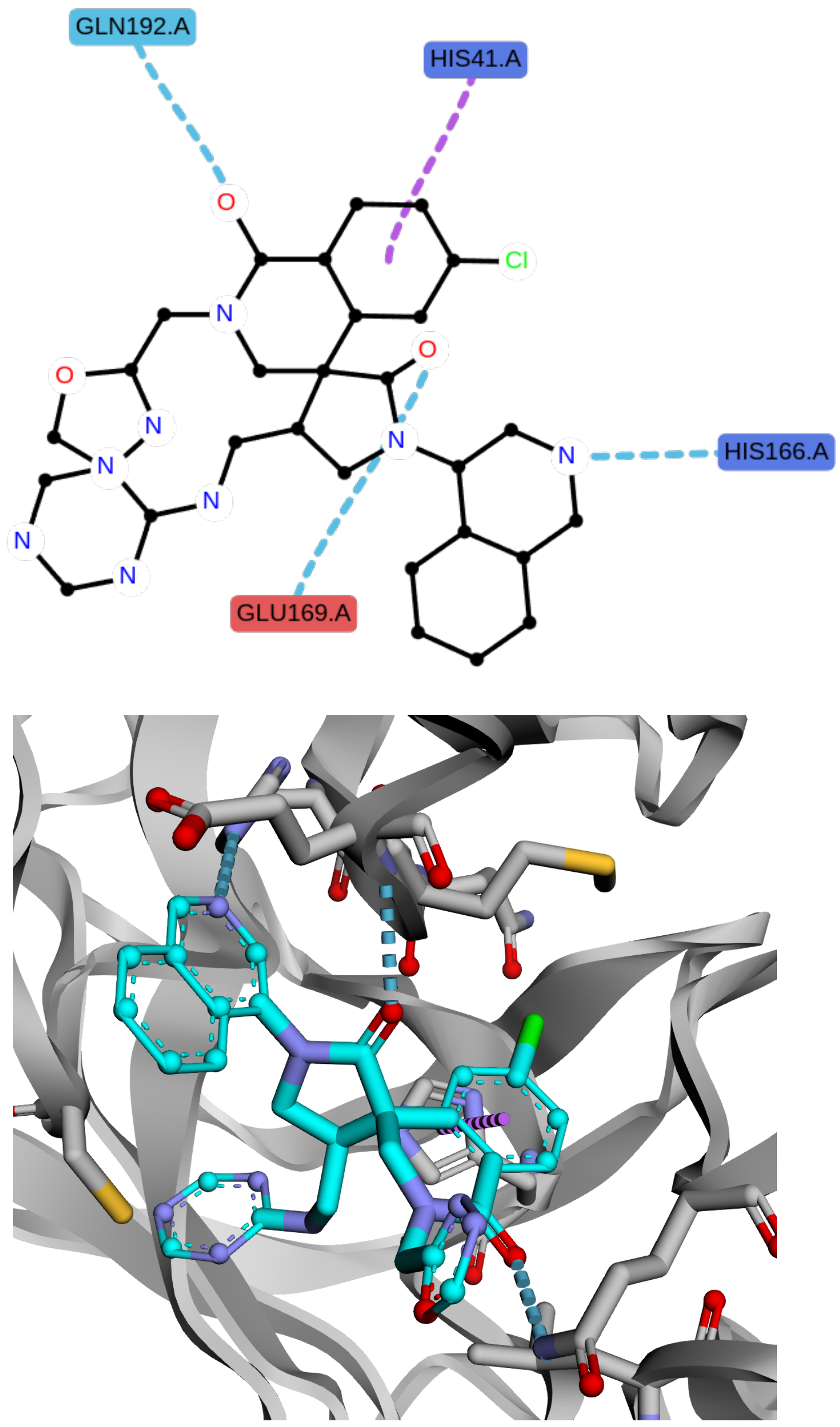

Figure S1: Example (top) 2D and (bottom) 3D protein-ligand interactions identified in the X-ray crystal structure of MERS-CoV Mpro bound to ligand 0 of the test set. Hydrogen bond acceptors are shown as dashed blue lines, and π-stacking in purple.

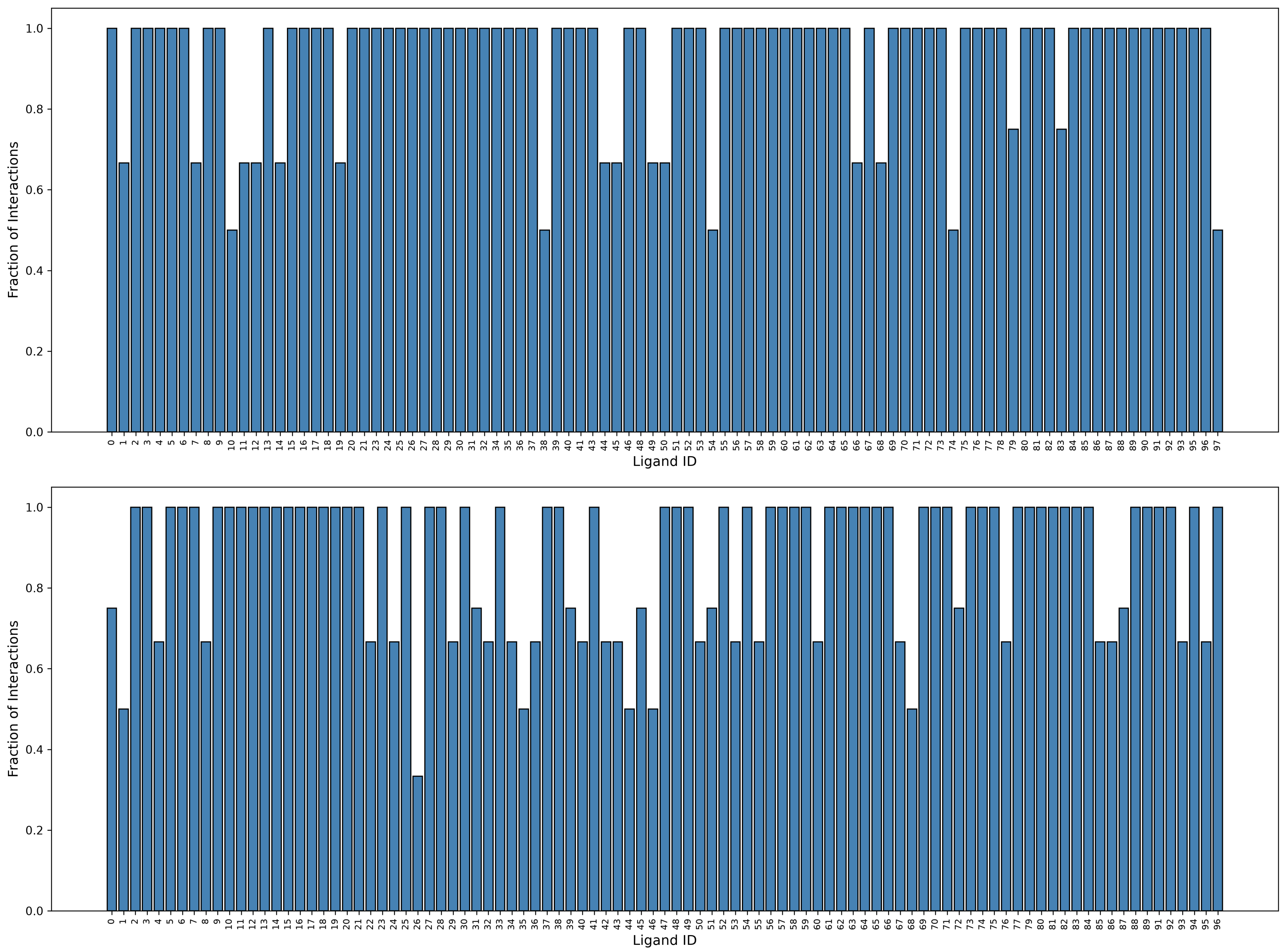

Figure S2: Maximum fraction of native X-ray crystal structure polar and π-stacking contacts present in at least one member of the computational ensemble generated by our retrospective workflow for (top) SARS-CoV-2 and (bottom) MERS-CoV Mpro.

Supplementary References

(1) Clark, F.; Robb, G. R.; Cole, D. J.; Michel, J. Automated Adaptive Absolute Binding Free Energy Calculations. *J. Chem. Theory Comput.* **2024**, *20*, 7806–7828.

(2) Clark, F.; Du, H. R. A3fe: Automated Adaptive Absolute Alchemical Free Energy Calculator. `https://zenodo.org/records/17298077`.

(3) Hedges, L.; Mey, A.; Laughton, C.; Gervasio, F.; Mulholland, A.; Woods, C.; Michel, J. BioSimSpace: An Interoperable Python Framework for Biomolecular Simulation. *JOSS* **2019**, *4*, 1831.

(4) Hedges, L. O.; Bariami, S.; Burman, M.; Clark, F.; Cossins, B. P.; Hardie, A.; Herz, A. M.; Lukauskis, D.; Mey, A. S. J. S.; Michel, J.; Scheen, J.; Suruzhon, M.; Woods, C. J.; Wu, Z. A Suite of Tutorials for the BioSimSpace Framework for Interoperable Biomolecular Simulation [Article v1.0]. *Living J. Comput. Mol. Sci.* **2023**, *5*, 2375–2375.

(5) Woods, C. J.; Hedges, L. O.; Mulholland, A. J.; Malaisree, M.; Tosco, P.; Loeffler, H. H.; Suruzhon, M.; Burman, M.; Bariami, S.; Bosisio, S.; Calabro, G.; Clark, F.; Mey, A. S. J. S.; Michel, J. Sire: An Interoperability Engine for Prototyping Algorithms and Exchanging Information between Molecular Simulation Programs. *J. Chem. Phys.* **2024**, *160*, 202503.

(6) Eastman, P.; Galvelis, R.; Peláez, R. P.; Abreu, C. R. A.; Farr, S. E.; Gallicchio, E.; Gorenko, A.; Henry, M. M.; Hu, F.; Huang, J.; Krämer, A.; Michel, J.; Mitchell, J. A.; Pande, V. S.; Rodrigues, J. P.; Rodriguez-Guerra, J.; Simmonett, A. C.; Singh, S.; Swails, J.; Turner, P.; Wang, Y.; Zhang, I.; Chodera, J. D.; De Fabritiis, G.; Markland, T. E. OpenMM 8: Molecular Dynamics Simulation with Machine Learning Potentials. *J. Phys. Chem. B* **2024**, *128*, 109–116.

(7) Maier, J. A.; Martinez, C.; Kasavajhala, K.; Wickstrom, L.; Hauser, K. E.; Simmerling, C. ff14SB: Improving the Accuracy of Protein Side Chain and Backbone Parameters from ff99SB. *J. Chem. Theory Comput.* **2015**, *11*, 3696–3713.

(8) Wang, J.; Wang, W.; Kollman, P. A.; Case, D. A. Automatic Atom Type and Bond Type Perception in Molecular Mechanical Calculations. *J. Mol. Graphics Modell.* **2006**, *25*, 247–260.

(9) Jorgensen, W. L.; Chandrasekhar, J.; Madura, J. D.; Impey, R. W.; Klein, M. L. Comparison of Simple Potential Functions for Simulating Liquid Water. *J. Chem. Phys.* **1983**, *79*, 926–935.

(10) Boothroyd, S.; Behara, P. K.; Madin, O. C.; Hahn, D. F.; Jang, H.; Gapsys, V.; Wagner, J. R.; Horton, J. T.; Dotson, D. L.; Thompson, M. W.; Maat, J.; Gokey, T.; Wang, L.-P.; Cole, D. J.; Gilson, M. K.; Chodera, J. D.; Bayly, C. I.; Shirts, M. R.; Mobley, D. L. Development and Benchmarking of Open Force Field 2.0.0: The Sage Small Molecule Force Field. *J. Chem. Theory Comput.* **2023**, *19*, 3251–3275.

(11) Jakalian, A.; Bush, B. L.; Jack, D. B.; Bayly, C. I. Fast, Efficient Generation of High-Quality Atomic Charges. AM1-BCC Model: I. Method. *J. Comput. Chem.* **2000**, *21*, 132–146.

(12) Jakalian, A.; Jack, D. B.; Bayly, C. I. Fast, Efficient Generation of High-Quality Atomic Charges. AM1-BCC Model: II. Parameterization and Validation. *J. Comput. Chem.* **2002**, *23*, 1623–1641.

(13) Shirts, M. R.; Chodera, J. D. Statistically Optimal Analysis of Samples from Multiple Equilibrium States. *J. Chem. Phys.* **2008**, *129*, 124105.

(14) Bieniek, M. K.; Cree, B.; Pirie, R.; Horton, J. T.; Tatum, N. J.; Cole, D. J. An open-source molecular builder and free energy preparation workflow. *Communications chemistry* **2022**, *5*, 136.

(15) Bouysset, C.; Fiorucci, S. ProLIF: a library to encode molecular interactions as fingerprints. *Journal of Cheminformatics* **2021**, *13*, 72.
